# Reciprocal transplants support a plasticity-first scenario during colonisation of a large hyposaline basin by a marine macro alga

**DOI:** 10.1186/s12898-017-0124-1

**Published:** 2017-04-05

**Authors:** Daniel Johansson, Ricardo T. Pereyra, Marina Rafajlović, Kerstin Johannesson

**Affiliations:** 1 Department of Marine Sciences, University of Gothenburg, Tjärnö, Strömstad Sweden; 2 Centre for Marine Evolutionary Biology, University of Gothenburg, Tjärnö, Strömstad Sweden; 3 Department of Physics, University of Gothenburg, Gothenburg, Sweden

**Keywords:** Common garden, Reciprocal transplant, Salinity, Asexual reproduction, *Fucus vecisulosus*, *Fucus radicans*, Baltic Sea

## Abstract

**Background:**

Establishing populations in ecologically marginal habitats may require substantial phenotypic changes that come about through phenotypic plasticity, local adaptation, or both. West-Eberhard’s “plasticity-first” model suggests that plasticity allows for rapid colonisation of a new environment, followed by directional selection that develops local adaptation. Two predictions from this model are that (i) individuals of the original population have high enough plasticity to survive and reproduce in the marginal environment, and (ii) individuals of the marginal population show evidence of local adaptation. Individuals of the macroalga *Fucus vesiculosus* from the North Sea colonised the hyposaline (≥2–3‰) Baltic Sea less than 8000 years ago. The colonisation involved a switch from fully sexual to facultative asexual recruitment with release of adventitious branches that grow rhizoids and attach to the substratum. To test the predictions from the plasticity-first model we reciprocally transplanted *F. vesiculosus* from the original population (ambient salinity 24‰) and from the marginal population inside the Baltic Sea (ambient salinity 4‰). We also transplanted individuals of the Baltic endemic sister species *F. radicans* from 4 to 24‰. We assessed the degree of plasticity and local adaptation in growth and reproductive traits after 6 months by comparing the performance of individuals in 4 and 24‰.

**Results:**

Branches of all individuals survived the 6 months period in both salinities, but grew better in their native salinity. Baltic Sea individuals more frequently developed asexual traits while North Sea individuals initiated formation of receptacles for sexual reproduction.

**Conclusions:**

Marine individuals of *F. vesiculosus* are highly plastic with respect to salinity and North Sea populations can survive the extreme hyposaline conditions of the Baltic Sea without selective mortality. Plasticity alone would thus allow for an initial establishment of this species inside the postglacial Baltic Sea at salinities where reproduction remains functional. Since establishment, the Baltic Sea populations have evolved adaptations to extreme hyposaline waters and have in addition evolved asexual recruitment that, however, tends to impede local adaptation. Overall, our results support the “plasticity-first” model for the initial colonisation of the Baltic Sea by *Fucus vesiculosus*.

**Electronic supplementary material:**

The online version of this article (doi:10.1186/s12898-017-0124-1) contains supplementary material, which is available to authorized users.

## Background

Some species are able to establish populations in ecologically marginal habitats where the physical environment is radically different from the species’ native environment. If a marginal habitat is relatively local, a population may be established and sustained by continuous recruitment of individuals from a nearby population forming a “source-sink” relationship [1]. However, some marginal environments are larger than the typical dispersal distance of a species, and populations established in these areas must be self-sustained. If this requires new phenotypic traits or phenotypic buffering [2], plasticity and/or directional selection need to be involved in the tuning of traits so that the individuals survive the new environment. Plasticity may seem to be the ideal mechanism, as colonisation in the presence of plasticity can take place without significant losses of genetic variation [3]. However, various constraints including increased costs of plasticity may put a halt to colonising new environments [4]. Moreover, plasticity may not always be adaptive and establishing a population in an environment outside the range of the native environmental variation may increase the risk of plasticity being harmful. If genetic variation is already present in a population as standing genetic variation, local adaptation by means of directional selection may be an efficient and rapid alternative to plasticity [5]. In comparison to plasticity, however, selection will, most likely, be accompanied by much larger loss of genetic variation. If new mutations are required, adaptation will be limited by the waiting time for these, which is usually very long [6]. Furthermore, local adaptation may be counteracted by gene flow [1], and restricted by demographic characteristics of species, such as small population sizes and long generation times [7]. On the other hand, it has been shown that hybridisation and introgression may contribute new genetic variation that may support local adaptation [8].

The Baltic Sea is one of the world’s largest brackish-water environments, and, as such, a truly marginal marine habitat. This postglacial semi-enclosed brackish water basin formed from a freshwater lake that opened to the sea about 8500 years ago [9]. Today the Baltic Sea has a surface salinity ranging from 2 to 3‰ in its innermost parts, 6–8‰ in the central parts, and towards the opening to the North Sea, through the Danish Straits, salinity changes rapidly from ~8 to ~20‰. Outside these straits, surface salinity successively increases to full marine salinity (>30‰) in the western part of the North Sea.

Since the opening, the Baltic Sea has been colonised by marine species, some of which have been introduced from other parts of the world [10], but the bulk of marine species have invaded the Baltic Sea from nearby areas of the North Sea. Following the North Sea-Baltic Sea transect, a majority of the marine species shows genetic clines that are steepest in the Danish Straits where the salinity gradient is strongest [11]. The steep clines are caused by local adaptation (e.g. [12, 13]), and isolation effects related to this.

Fucoid macroalgae are foundation species of rocky-shore ecosystems in temperate and subarctic waters. Of a handful of Atlantic species, one species, *Fucus vesiculosus*, has been able to establish populations deep inside the Baltic Sea including areas of strong hyposaline (2–4‰) waters, such as the inner parts of the Gulf of Riga, the Gulf of Finland and the Bothnian Sea [14, 15]. A most intriguing finding is that the colonisation of the Baltic Sea is paralleled by a switch from what seems to be obligate sexual recruitment of new attached thalli outside the basin, to facultative asexual recruitment of new attached and fully sexually reproduced thalli inside the basin [16]. It has been suggested that this switch is due to an increased risk of polyspermy and failure of sexual reproduction in hyposaline waters [17]. However, predominance of recruitment by cloning is not everywhere correlated to salinity [18]. During establishment of *F. vesiculosus* in the Baltic Sea, a separate species (*F. radicans*) diverged from the Baltic lineage of *F. vesiculosus* [19, 20]. *Fucus radicans* is endemic in the Baltic Sea. It is sympatric with *F. vesiculosus* over large parts of the Bothnian Sea and in Estonia [15, 21]. Both species are dioecious with both males and females being capable of asexual reproduction.

Asexual reproduction in Baltic Sea populations of both species is accomplished by the production of adventitious branches that come loose and reattach to the bottom by formation of rhizoids [16]. Adventitious branches are present also in thalli of *Fucus* outside the Baltic Sea, where they have most likely been formed after physical damage from grazers [22], but asexual formation of new thalli has never been reported outside the Baltic Sea.

We hypothesised that the establishment in the Baltic Sea by *F. vesiculosus* may have followed the plasticity-first model [3]. We investigated this by testing two predictions from the model: (i) individuals of the original population have high enough plasticity to tolerate the marginal environment, and (ii) individuals of the marginal population show evidence of local adaptation. The Baltic Sea populations of *F. vesiculosus* and *F. radicans* both descend from a common *F. vesiculosus* lineage originating in the eastern part of the North Sea, close to the entrance of the Baltic Sea [21]. Thus we used individuals from a population in this area to represent the ancestors from which the current Baltic Sea individuals of both species have derived. We used a reciprocal transplant experiment [23] to compare the development of key traits between North Sea and Baltic Sea populations in both native and non-native salinity. This allowed us to separate between locally adapted and plastic traits. To avoid confounding effects from the native environment we used small adventitious branches that were detached from the mother thalli and cultured during 6 months in the laboratory. We assessed survival and measured growth rate as proxies for physiological tolerance and general fitness. We also recorded formation of a second generation of small adventitious branches from the first generation branches and the formation of rhizoids from the primary branches. Both formation of adventitious branches and formation of rhizoids are necessary to accomplish asexual reproduction. Finally, we recorded formation of receptacles as a proxy for the allocation of resources into sexual reproduction.

## Methods

### Sampling and characterisation

**Fig. 1:**
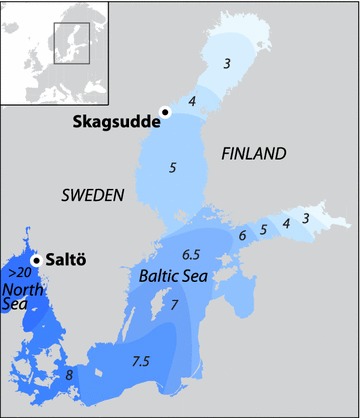
Map showing sampling sites and the Baltic Sea salinity gradient. Source: Online Map Creation-Martin Weinelt, http://aquarius.ifm-geomar.de, visited 2009.05.01

We sampled *F. radicans* and *F. vesiculosus* (73 individuals in total) from a sympatric site on the Swedish coast of the northern Baltic Sea (Skagsudde, N 63°11′21″, E 19°0′13″; Fig. 1). In addition, we sampled 20 individuals of *F. vesiculosus* from a North Sea population on the Swedish west coast (Saltö, N 58°52′16″, E 11°7′11″; Fig. 1). The Baltic samples were from a depth of 3–6 m and an average salinity of 4‰, while the North Sea samples were from the intertidal, with fluctuating salinity around an average of 24‰ (range 15–30‰). Sampling was performed in July 2011 and fresh thalli were brought to the laboratory and stored in tanks with water of ambient salinity (4 and 24‰, respectively). We hereafter refer to each of these three samples (*F. radicans* from the Baltic Sea, *F. vesiculosus* from the Baltic Sea, and *F. vesiculosus* from the North Sea) as our three “populations”. Formal identification of the individuals within each sample was done by D.J. on basis of morphological criteria and microsatellite genotype.

For the two Baltic Sea species we performed a first separation based on morphology and confirmed (or in a few cases, corrected) the identification using genotypes in 9 microsatellite markers. Extraction of DNA, PCR reactions and microsatellite analyses were done following the description in [24]. The software STRUCTURE [25] was used to assign individuals into genetically coherent groups, and GENCLONE [26] was used to identify clones among the Baltic Sea individuals. All individuals from the North Sea, on the other hand, were a priori assumed to be unique multi-locus genotypes as asexual reproduction has never been reported from outside the Baltic Sea [16, 27].

We assessed background phenotypic differentiation between Atlantic and Baltic Sea populations by measuring variation in morphological traits in all thalli sampled in the field using the traits earlier described to discriminate between the two species [19]. The measurements included distance between the two most distant dichotomies on a branch, frond width measured midway between the same two dichotomies (both these measurements were repeated in five branches per individual), stipe length measured as the distance between the holdfast and the first branching point, and total length measured as the distance between the holdfast and the most distal tip. We used principal components analysis to assess overall differences among the three populations in size and shape.

In a long-term (6 months) reciprocal transplant experiment North Sea and Baltic Sea adventitious branches detached from large thalli grew new vegetative tissue in both 4 and 24‰ salinity. In this experiment each individual was represented by six ≈1 cm long adventitious branches that we randomly chose from a large number of adventitious branches grown on each individual. We used 48 individuals of Baltic *F. radicans*, 25 of Baltic *F. vesiculosus* and 20 of North Sea *F. vesiculosus*. Three branches from each individual were acclimatised to the low salinity and three to the high, by adjusting the salinity gradually over 4 weeks for those transplanted to a non-native salinity. The adventitious branches were incubated upright, attached with elastic threads to holders in tanks with 40 L water and flow-pumps to circulate the water. We used two tanks per salinity and individuals were randomly distributed between these while keeping numbers of individuals from each population constant in each tank. The water was prepared by mixing tap water with “Instant Ocean” salt and nutrient medium. Water was changed once every 2nd month. The experiment was run in a thermo constant room at 13 °C and a 16:8 h light: dark cycle for 6 months. The length increment of each adventitious branch was measured at the end of the experiment. In addition, we counted the number of new adventitious branches (“secondary branches”) formed from the starting branches. Rhizoids (used to attach the vegetative part to the substratum) were formed at the basal end of some of the primary adventitious branches, and we noted presence or absence of rhizoids in any of the three replicate branches of the same individual after inspection using a stereomicroscope. Some of the primary adventitious branches also formed early stages of receptacles and in a similar way, we recorded presence or absence of these in each individual.

### Statistical analysis

We conducted two separate analyses for each response parameter. In one analysis we compared the two sympatric populations of *F. radicans* and *F. vesiculosus* from the Baltic Sea. In a separate analysis we compared the Baltic and North Sea populations of *F. vesiculosus*. For all tests including *F. radicans* we performed analyses with both the full dataset, and datasets that were made balanced (*N* = 20) by random removal of individuals, using type-III sums of squares in the unbalanced cases. As results obtained in balanced and full data sets remained qualitatively the same, we only present the result of the balanced data set in figures and tables.

As adventitious branches of each individual were grown in both salinities (see above), we used a split-plot design for the analysis of variance (ANOVA). We assessed assumptions of normality and homogeneity of variances with box-plots and residual plots. Log transformation improved the growth data, which was analysed using the *aov* function in R (v. 2.15.2; [28]). Data on formation of secondary adventitious branches were analysed as a split-plot design with linear mixed-effects models using the *lme* function. We analysed occurrence (presence/absence) of rhizoids using Fisher’s exact test, comparing species and origin separately. As rhizoids only formed in the high salinity in all three populations, the low salinity data was not included in the analysis. Receptacles were only formed by one population and in one salinity treatment, and these results were left without statistical evaluation. Confidence intervals for all means were calculated by multiplying standard error with the critical t-value at a confidence level of 95%.

## Results

In the Baltic Sea site where both *F. radicans* and *F. vesiculosus* were sampled, the preliminary assignment of individuals to species, based on overall morphology, was mostly consistent with the result of the genetic analysis. Among the individuals sampled, however, four individuals were not correctly assigned from morphology and were moved to the correct taxa for all down-stream analyses after genotyping (Additional file 1). The genotyping further showed that the Baltic population of *F. vesiculosus* consisted of 10 unique multi-locus genotypes and 7 clones with 2–3 individuals per clone (in total 17 genotypes), while the population of *F. radicans* included 2 unique individuals and three clones with 9, 17 and 20 individuals each (in total 5 genotypes). Despite the replication of genotypes, in particular in *F. radicans*, we decided to include all individuals sampled in the experiment, in order to form a representative sample of a wild population from the Baltic Sea and to detect (if present) contributions from somatic mutations. Separate analyses were in addition done comparing traits among the three major clones of the *F. radicans* samples (see below).

**Fig. 2:**
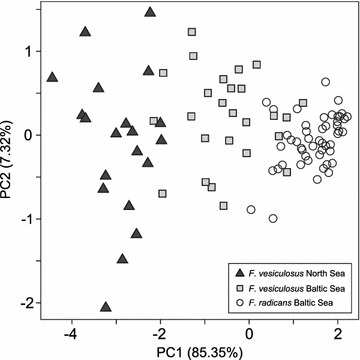
Principal components analysis of four morphological characters (see text) in adult populations of *F. vesiculosus* from Saltö (North Sea), *F. vesiculosus* from Skagsudde (Baltic Sea) and *F. radicans* from Skagsudde (Baltic Sea)

The morphology of the adult thalli sampled in the wild revealed phenotypic variation among the three populations along the PC1 axis, reflecting population differences in overall size. Individuals of *Fucus vesiculosus* from the North Sea were, on average, larger than individuals of Baltic Sea *F. vesiculosus*, and individuals of *F. radicans* were the smallest (Fig. 2). There was no separation of the populations along the PC2 axis and this suggests that there were no major differences in shape among the three populations. The within population variation along the PC2 axis tended to be highest in the North Sea *F. vesiculosus* and lowest in the *F. radicans* population.

**Fig. 3:**
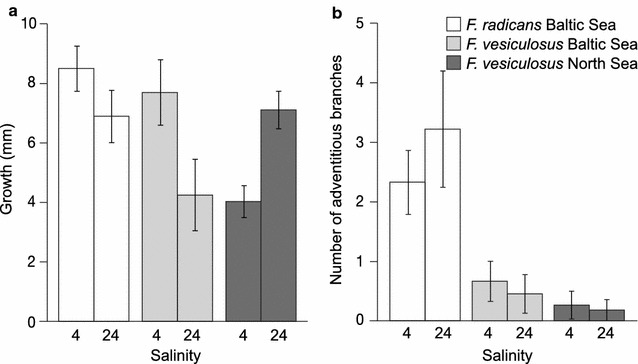
Result of reciprocal transplants of North Sea and Baltic Sea *Fucus* during 6 months in 4 and 24‰ salinity. **a** Growth of 10 mm large adventitious branches. **b** Number of secondary branches formed per primary branch. Error bars show 95% CI

**Fig. 4:**
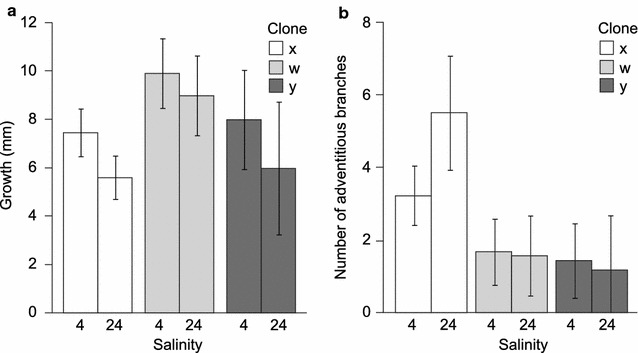
The same experiment as in Fig. 3, comparing the results for the three large clones of *Fucus radicans*. N = 20 for clone X, 17 for W and 9 for Y. **a** Growth of 10 mm large adventitious branches. **b** Number of secondary branches formed per primary branch. Error bars show 95% CI. (For statistic evaluation see Tables 2 and 4.)

Among the adventitious branches transferred to a non-native salinity (from 4 to 24‰, or the reverse), 1–3 (and usually 3) of the replicate branches of each individual survived and formed new tissue during the 6 months experiment, indicating high plasticity in the physiological response to environmental salinity. However, comparing growth of Baltic and North Sea *F. vesiculosus*, we found a strong interaction between salinity and origin (*P* < 0.001; Table 1), indicating that both populations of *F. vesiculosus* grew better in their native salinity than in the other salinity. This supports that Baltic Sea populations have evolved local adaptation in traits that contribute to growth (Fig. 3a). Also the Baltic Sea population of *F. radicans* grew better in the hyposaline conditions of its native environment than in fully marine waters (Fig. 3a). Notably, *F. radicans* was less negatively impacted by high salinity than was Baltic *F. vesiculosus*, resulting in a significant interaction be-tween salinity and species also in this comparison (*P* = 0.033; Table 1; Fig. 3a). We also compared growth rates among the three numerically dominant clones of *F. radicans* from the Baltic Sea and found a statistically significant effect of clone on growth with a similar trend of higher growth in the low salinity in all clones (Table 2; Fig. 4a), supporting the presence of genetic variation in this trait.

**Table 1:** ANOVA (split-plot design) analysing growth of adventitious branches during 6 months in a reciprocal transplant experiments as an effect of salinity (4 and 24‰), species and origin

Source	d*f*	*SS*	*MS*	*F*	*P*
A. Baltic Sea *F. radicans* vs. *F. vesiculosus*					
Species	1	0.688	0.688	2.191	0.148
Residuals	36	11.312	0.314		
Salinity	1	4.782	4.782	35.67	<0.001
Species*salinity	1	0.662	0.662	4.939	0.033
Residuals	36	4.826	0.134		
B. Baltic Sea *F. vesiculosus* vs. North Sea *F. vesiculosus*					
Origin	1	<0.001	<0.001	<0.001	0.985
Residuals	36	10.62	0.295		
Salinity	1	0.052	0.052	0.375	0.544
Origin*salinity	1	7.689	7.689	55.757	<0.001
Residuals	36	4.964	0.138		

A. Comparison of growth rates between *F. radicans* and *F. vesiculosus* from a sympatric site in the Baltic Sea. B. Comparison of growth rates between *F. vesiculosus* from North Sea and Baltic Sea. See also Fig. 3a

**Table 2:** ANOVA (split-plot design) analysing growth of adventitious branches during 6 months in a reciprocal transplant experiments as an effect of salinity (4 and 24‰), among three clones of *Fucus radicans* from Skagsudde (Baltic Sea), see also Fig. 4a

Source	*df*	*SS*	*MS*	*F*	*P*
Clone	2	167.9	83.97	7.57	0.002
Residuals	43	476.9	11.09		
Salinity	1	54.7	54.72	16.45	<0.001
Clone*Salinity	2	5.22	2.61	0.784	0.46
Residuals	43	143.1	3.33

Secondary adventitious branches were formed from the basal part of the original adventitious branches in all three populations during the experimental period. When we compared the two populations of *F. vesiculosus* from the North Sea and the Baltic Sea, respectively, we found no significant effect of salinity and no interaction between salinity and origin, but a trend towards higher numbers of secondary adventitious branches in the Baltic population than in the North Sea population (*P* = 0.073; Table 3; Fig. 3b). Comparing the two sympatric populations from the Baltic Sea, *F. radicans* and Baltic *F. vesiculosus*, we found no significant interaction of species and salinity, and no significant effect of salinity alone. However, there was a strong difference between the two species (*P* < 0.001, Table 3), that is, *Fucus radicans* formed significantly more secondary adventitious branches than did sympatric individuals of *F. vesiculosus* in both salinities (Fig. 3b). Also in this trait we found differences among clones with one clone growing more adventitious branches than the other two clones, showing genetic variation being present in this trait (Table 4; Fig. 4b).

**Table 3:** ANOVA (linear mixed effects model, d*f* = 1.34) analysing the number of secondary formed adventitious branches per primary branch, during 6 months in a reciprocal transplant experiments, as an effect of salinity (4 and 24‰), species and origin

*Source*	*F*	*P*
A. Baltic Sea *F. radicans* vs. *F. vesiculosus*		
Intercept	36.45	<0.001
Salinity	0.876	0.36
Species	15.65	<0.001
Salinity*species	2.648	0.11
B. Baltic Sea *F. vesiculosus* vs. North Sea *F. vesiculosus*		
Intercept	24.74	<0.001
Salinity	1.989	0.17
Origin	3.42	0.07
Salinity*origin	0.261	0.61

A. Analysing the effect of salinity and species (*F. radicans* and *F. vesiculosus)* from the same origin in the Baltic Sea. B. Analysing the effect of salinity and origin (North Sea and Baltic Sea) in populations of *F. vesiculosus.* See also Fig. 3b

**Table 4:** Linear mixed-effects model of number of secondary adventitious branches formed by the three clones of *Fucus radicans* from Skagsudde during growth in a common garden at two different salinities (see also Fig. 4b)

	d*f*	*F*	*P*
Intercept	1.43	136.8	<0.001
Salinity	1.43	0.049	0.83
Clone	2.43	15.63	<0.001
Clone*salinity	2.43	3.24	0.049

Some of the primary adventitious branches developed rhizoids and attached to the substratum, but unexpectedly rhizoids were only formed in the higher salinity treatment (24‰). The proportion of individuals that developed rhizoids was different among the three populations with 61% of *F. radicans* individuals and 20% of Baltic Sea *F. vesiculosus* forming rhizoids, but none of the North Sea *F. vesiculosus* did form rhizoids. In the sympatric Baltic Sea site, the difference between the two species was significant, and the difference between *F. vesiculosus* of Baltic Sea and North Sea origin was in addition marginally significant (Table 5). There was, however, no significant difference in how many individuals formed rhizoids among the three clones of *F. radicans* (results not shown). Although not quantified, we observed that most of the adventitious branches that formed rhizoids also attached to the substratum.

**Table 5:** Proportion of individuals forming rhizoids in the three study populations of *Fucus*

Baltic Sea		North Sea
*Fucus radicans*	*F. vesiculosus*	*F. vesiculosus*
61%	20%	0%
*P* = 0.003		
	*P* = 0.053	

*P* values are derived from Fisher’s exact test

In 35% of the North Sea individuals the adventitious branches formed early stages of receptacles in the high salinity treatment. No receptacles were developed in the adventitious branches from the two Baltic populations in high or low salinity, or in adventitious branches of the North Sea population kept in low salinity.

## Discussion

West-Eberhard [3] suggests that developmental plasticity is likely to precede the accumulation of genetic divergence during colonisation of a new environment. The mechanism is simply that a population in which all individuals survive the new environment by being plastic will avoid demographic bottlenecks during establishment caused by selective mortality. Following successful colonisation, assimilation of genetic differences that improves phenotypes and result in local adaptation of traits may follow [3]. Here we raise the question if the colonisation of the Baltic Sea by *F. vesiculosus* was initially made possible by a high degree of plasticity, but later followed by assimilation of genetic differences caused by directional selection that improved local adaptation and allowed for further colonisation up to the current range margin in extreme hyposaline waters. The plasticity-first model is supported if colonising individuals without prior adaptation will survive and reproduce in the new environment due to their plasticity. In contrast to a model where colonisation would only be possible under selective mortality, establishment aided by plasticity would give less serious demographic effects and probably less loss of genetic variation by selection and drift, although plasticity would mask the genetic variation present in traits and may prevent improvements in fitness by local adaptation.

We here show that contemporary individuals of the North Sea population of *F. vesiculosus* have a high tolerance to low salinity and survive and grow in strongly hyposaline (4‰) waters. Assuming a similar capacity in the North Sea population during the time of the Baltic Sea invasion of *F. vesiculosus*, the individuals that colonised the Baltic Sea were capable of surviving at salinities currently found at the range margin of the Baltic *Fucus* distribution. However, for successful establishment in the Baltic Sea without prior local adaptation, also traits central to reproduction must remain functional in the low salinity. Indeed, an earlier study of sexual reproduction shows that gametes of *F. vesiculosus* from the North Sea remain functional down to salinities around 7–8‰ [17]. Thus high levels of plasticity in survival, growth and sexual function suggest that populations from the North Sea were able to rapidly colonise the southern part of the hyposaline Baltic Sea after its postglacial formation, or even more central parts as salinity during a period around 6000 years ago was higher than today [9]. Due to the lack of selective mortality this phase of the colonisation was presumably relatively rapid, and without major losses of genetic variation. Following the establishment in the southern and central parts of the Baltic Sea, the population seems to have improved its fitness by directional selection. The selection led to local adaptation and higher growth rate in 4‰ compared to 24‰ salinity, as shown in this study (and see [29]). Also local adaptation in sexual reproduction has taken place as current Baltic Sea populations maintain high sperm quality and rate of fertilisation below 8‰ [17]. There is also support for local adaptation in other traits of Baltic Sea *F. vesiculosus* and *F. radicans*. For example, tolerance to emersion stress is lost in Baltic Sea populations following the switch to a more subtidal distribution [30], and a majority of genotypes in *F. radicans* have reduced tolerance to stress from desiccation and freezing [31]. Thus following the initial phase of establishment in the Baltic Sea supported by plasticity, the second phase involving selection towards increased fitness in hyposaline waters seems also to have extended the distribution of the *F. vesiculosus* lineage (including the *F. radicans* branch) to its current range margins in extremely low salinities (3–4‰).

As part of the second phase of local adaptation, the new role of the adventitious branches is intriguing. The original (North Sea) role of these vegetative parts was probably to repair tissue damaged by grazers [22]. In the northern Baltic Sea, by contrast, adventitious branches form, fall off and develop rhizoids that reattach them to the substratum, and from there they grow clonal copies of the mother thallus. Formation of adventitious branches was most frequent in *F. radicans*, less frequent in the Baltic Sea *F. vesiculosus*, and least common or absent in North Sea *F. vesiculosus* in our experiments, which correlates to the prevalence of clones in these populations (this study and [32]). Asexual reproduction by means of re-attaching adventitious branches seems to be a unique trait to the Baltic Sea that in itself has promoted the spread and establishment in a new area. The reason for this may be that after its initial occurrence, asexual reproduction may have spread in the northern Baltic Sea by directional selection favouring individuals that invested more in asexual than in sexual reproduction. The observation of polyspermy in salinities below 5–6‰ [33] supports a selection-driven switch to asexual reproduction in hyposaline waters. Polyspermy may severely constrain sexual reproduction, at least in populations where gamete densities are high. Under risk of polyspermy, individuals that allocate more energy to formation of adventitious branches and less to building receptacles and forming gametes would be favoured by selection.

However, in the field also thalli recruited asexually form receptacles and produce gametes that are functional [16, 34]. This suggests that selection for asexual reproduction in hyposaline areas is not particularly strong. In addition, the observation that the prevalence of asexual recruitment is very variable and not correlated to salinity [18] suggests directional selection imposed by salinity being a less likely explanation for the spread of asexual recruitment inside the Baltic Sea. Alternatively, the distribution of clones may be a consequence of neutral processes during colonisation of the Baltic Sea (that is, random dispersal and demographic stochasticity). Indeed, according to recent modelling results, it seems highly likely that if asexual reproduction is an option, then fully neutral processes are likely to drive structuring of the population and determine the distribution of asexual populations during a colonisation event without involvement of selection on individual reproductive fitness [35].

## Conclusions

*Fucus vesiculosus* is the only Atlantic lineage of fucoid algae that has been able to colonise deep into the Baltic Sea including environments with strong hyposaline conditions. As shown in this study, North Sea individuals of this species are tolerant enough to survive at least several months in salinities corresponding to the hyposaline conditions at the range margin of Baltic Sea populations. Moreover, as shown in an earlier study [17], sexual reproduction in North Sea *F. vesiculosus* is sustained down to salinities typical of the central Baltic Sea. Thus, plasticity in growth and sexual reproduction seemed strong enough to allow for an initial colonisation phase into the central Baltic Sea without selective mortality, corroborating the primary prediction of the plasticity-first model of West-Eberhard [3]. Furthermore, growth of North Sea individuals is already plastic enough to sustain survival down to strongly hyposaline waters at the current Baltic Sea range margins, and this trait was thus “preadapted” to colonise deeper into the Baltic Sea upon arrival of the first populations. Sexual reproduction, on the other hand, was only enough plastic to sustain recruitment into the central Baltic Sea, but a dramatic switch to asexual reproduction most likely boosted further colonisation of more marginal areas. Both strong plasticity and dominance of asexual recruitment somewhat impede the action of selection. But, nevertheless, the Baltic Sea *Fucus* shows evidence of local adaption in several key traits supporting the second prediction of the plasticity-first hypothesis that the initial phase of colonisation is followed by improved fitness and local adaptation through directional selection [3].

## Additional file

**Additional file 1.** Result of structure analysis. This analysis was used to confirm species assignment in the sympatric sample of *Fucus radicans* and *F. vesiculosus* from the Baltic Sea site.
