# Supplementary material for: Reciprocal transplants support a plasticity-first scenario during colonisation of a large hyposaline basin by a marine macro alga
Source: BMC Ecol. 2017 Apr 5;17:14. doi: 10.1186/s12898-017-0124-1 (PMC5382403; doi:10.1186/s12898-017-0124-1)
Supplement: Supplementary file 1 — Additional file 1. Result of structure analysis. This analysis was used to confirm species assignment in the sympatric sample of Fucus radicans and F. vesiculosus from the Baltic Sea site. [file 12898_2017_124_MOESM1_ESM.docx]

Additional file 1. **Result of structure analysis.** This analysis was used to confirm species assignment in the sympatric sample of *Fucus radicans* and *F. vesiculosus* from the Baltic Sea site.

STRUCTURE analysis based on microsatellite genotypes confirming species identify of *Fucus radicans* (green and yellow) and *F. vesiculosus* (blue and red) from a sympatric site, Skagsudde, in the Baltic Sea. Each vertical bar represents one individual. K (number of groups) is 4. The asterisks under the X-axis indicate three individuals that were erroneously assigned to *F. radicans* based on morphology. The °-symbol indicates one individual that was er­roneously assigned to *F. vesiculosus* based on morphology. These species identification of these four individuals were corrected in down-stream analyses.
